# Pharmacological Properties and the Impact of Caffeic Acid-Entrapped Liposomes on Triple-Negative Breast Cancer Cell Lines Exposed to Doxorubicin

**DOI:** 10.3390/antiox15040424

**Published:** 2026-03-27

**Authors:** Ioana-Lavinia Dejeu, Diana Olteanu, Simona Clichici, Ioana Baldea, Olga Soritău, Olimpia-Daniela Frenț, Nicole Alina Marian, Mariana Eugenia Mureșan, Paula Svera, Eleonora Marian, George Emanuiel Dejeu, Laura Grațiela Vicaș, Gabriela Adriana Filip

**Affiliations:** 1 Department of Pharmacy, Faculty of Medicine and Pharmacy, University of Oradea, No. 29 Nicolae Jiga Street, 410028 Oradea, Romania; ioana.dejeu@csud.uoradea.ro (I.-L.D.); ofrent@uoradea.ro (O.-D.F.); emarian@uoradea.ro (E.M.); 2 Department of Physiology, ‘‘Iuliu Hațieganu” University of Medicine and Pharmacy, 1-3 Clinicilor Street, 400006 Cluj-Napoca, Romania; diana.olteanu@iocn.ro (D.O.); sclichici@umfcluj.ro (S.C.); ioana.baldea@umfcluj.ro (I.B.); 3 Radiobiology and Tumor Biology Laboratory, Oncological Institute “Prof. Dr. I. Chiricuță”, 34–36 Republicii Street, 400015 Cluj-Napoca, Romania; olgasoritau@yahoo.com; 4 Doctoral School of Biomedical Sciences, University of Oradea, No. 1 University Street, 410087 Oradea, Romania; marian.nicolealina@student.uoradea.ro (N.A.M.); mmuresan@uoradea.ro (M.E.M.); 5 National Institute for Research and Development in Electrochemistry and Condensed Matter, 144th Dr. A.P. Podeanu Street, 300569 Timisoara, Romania; paula.svera@incemc.ro; 6 Department of Surgical Disciplines, Faculty of Medicine and Pharmacy, University of Oradea, 10 Piata 1 Decembrie Street, 410073 Oradea, Romania; dejeu.george@uoradea.ro; 7 Department of Anatomy and Embryology, “Iuliu Hatieganu” University of Medicine and Pharmacy, no 8 Victor Babeș Street, 400012 Cluj-Napoca, Romania; gabriela.filip@umfcluj.ro

**Keywords:** caffeic acid, liposomes, doxorubicin, breast cancer cell, oxidative stress

## Abstract

**Background:** Caffeic acid (CA), with antioxidant and immunomodulatory properties, was formulated in liposomes to increase its efficacy. The study targets triple-negative breast cancer (TNBC), characterized by the absence of ER, PR, and HER2 receptors. **Methods:** For CA-loaded liposomes, the pharmacological effects on TNBC cell lines, parental Hs578T (HS) and Doxorubicin-resistant Hs578T (HSD) cells were evaluated by determining the cell growth inhibition ratio measured by the (3-[4,5-dimethylthiazol-2-yl]-2,5 diphenyl tetrazolium bromide) assay, oxidative stress, apoptosis rate, membrane damage and transcription factor expressions, and DNA damage, with or without exposure to Doxorubicin (Dox). **The Results:** demonstrated that CA-loaded liposomes were stable and had high entrapment capacity. They exerted apoptotic effects on both cells, comparable to Doxorubicin, and increased cell membrane damage. The liposomes increased STAT3 expression in HS cells, while they reduced NRF2 and STAT3 in HSD cells, suggesting beneficial effects on Dox-resistant breast tumor cells. In HS cells exposed to Dox, CA treatment improved the number of viable tumor cells and decreased the rate of apoptosis, while in HSD cells it enhanced apoptosis as a mechanism of cell death and decreased pro-survival molecules, STAT3 expression in parallel with reduced NRF2 activation. **Conclusions:** The results indicated that CA encapsulated in liposomes was able to interfere with some survival mechanisms of triple-negative cells and could inhibit their proliferation.

## 1. Introduction

Liposomes have been used as carrier systems since 1960s in various fields including chemistry, mathematics, physics, biophysics and biology. Since then, their use has experienced a remarkable development and they are now being used in modern technology for the transport of active molecules to the target site. Liposomes contain phospholipids, the main components of cell membranes, which have both hydrophilic and hydrophobic parts. The hydrophilic component consists of choline, a phosphate group and glycerol, while the hydrophobic part contains two tails with essential fatty acids [1].

In medicine, the use of liposomes for controlled release of different drugs is now having an increasing impact. They have been shown to be beneficial for the stabilization of therapeutic compounds due to efficient release of encapsulated compounds to target organs, with low systemic toxicity.

Caffeic acid (CA) belongs to the class of polyphenols; it possesses important antioxidant and immunomodulatory activities [2], can induce antibacterial effect [3] and has beneficial effects in the prevention and adjuvant treatment of cancer [4].

In vitro study on human breast adenocarcinoma line has been shown that CA can act as apoptotic trigger in tumor cells and induce cell cycle arrest in G0/G1 phase [5]. Additionally, CA decreased the viability and migration of MDA-MB-231 human breast cancer cells [6]. Based on these properties, several studies have focused on finding formulas that improve and increase its effectiveness on the target cells, and thus the sensitivity of tumor cells to chemotherapy and radiotherapy [7]. In this context, formulation of CA in liposomes was tested as an option in chemoprevention and antiproliferative adjuvant treatment in oncology due to high stability, efficient cytotoxic effects on tumor cells and significant protection of normal cells [8].

Doxorubicin (Dox) is an anthracycline antibiotic, an effective antitumor drug, successfully used in various treatments for breast and lung cancers, acute leukemia, multiple myeloma and non-Hodgkin lymphomas [9,10,11,12]. The antitumoral effect of Dox imply DNA intercalation, inhibition of topoisomerase II and the formation of reactive oxygen species (ROS) with cytotoxic effect on tumor cells [13]. At mitochondrial membrane level, Dox binds specifically to inner mitochondrial membrane and inhibits ubiquinone oxidoreductase (complex I) and succinate dehydrogenase (complex II) from the electron transport chain, generating ROS. Overproduction of ROS and a reduced antioxidants defense, trigger lipid peroxidation, membrane and DNA damage and consequently induce apoptosis [14,15]. Frequently, Dox treatment was associated with some side effects such as cardiotoxicity, myelosuppression and resistance to therapy [16,17], especially in certain environmental conditions. Therefore, finding new treatments with selective cytotoxicity and additive effects on cancer cells, with minimal toxicity on normal tissues, represents an important strategy in oncology.

Based on these data, in our study, after the preparation and characterization of liposomes with CA, their biological effects on two types of mammary cancer cells treated with Dox were evaluated. Oxidative stress, cell membrane damage as well as DNA lesions and transcription factors expression were quantified to elucidate the mechanism of action of liposomes with CA in cells exposed or not to Dox. For testing, two triple-negative breast cancer cell lines, parental Hs578T (HS) and Hs578T cells resistant to Dox (HSD) were chosen. These cells are immunohistochemically characterized by the lack of the three receptors, estrogen (ER), progesterone (PR) and human epidermal growth factor receptor 2 (HER2), by a marked phenotypic aggressiveness, high metastatic ability, lack of response to treatment and unfavorable prognostic rate [18,19,20]. The key features of the behavior of these cells are related to alteration of several molecular pathways including cell cycle, DNA repair, NF-κB signaling, inflammatory response and angiogenesis [21]. Therefore, finding new compounds that interfere with these survival mechanisms is welcome and offers premises for overcoming resistance to therapy of aggressive tumors. This is the first formula developed for targeted CA delivery in triple-negative cells exposed to Dox, as functionalized CA-loaded liposomes have not yet been reported.

## 2. Materials and Methods

### 2.1. Preparation of Liposomes with CA and Without CA

The lipid film hydration method was used for the preparation of liposomes as a previously published method [22,23].

Four liposomes, two loaded with CA (DPPC, CNA) and two without CA (eDPPC, eCNA), were characterized for using in biological experiments. In order to detect the variation in their characteristics over time, at different time periods, the liposomes were analyzed as follow: day 1 (d1), day 2 (d2), day 15 (d15) and day 30 (d30), following previously published work [22,23]. The quantities of substances required for liposome synthesis are given in Table 1.

Physico-chemical characterization of four synthesized liposomes was performed using dynamic light scattering method (DLS) to evaluate the size and zeta potential, atomic force microscopy (AFM) to analyze liposome morphology, efficiency of CA entrapment in liposomes, and in vitro release of CA from liposomes (DPPC, CNA), according to previous studies [22,23].

The DLS (DLS ZEN 3690) and the Zetasizer Nano ZS (Malvern Panalytical, Malvern, Worcestershire, UK) were used to measure the size and zeta potential of the four formulated liposomes. The method used for measurement liposomes size was in agreement with those previously published [24,25,26]. All the measurements were performed in triplicate.

Scanning Probe Microscopy Platform (MultiView-2000 system, Nanonics Imaging Ltd., Jerusalem, Israel) with intermittent mode was used in ambient conditions (20 °C) to perform the morphological analyzes. A scanner equipped with a silicone probe and covered with chrome was used, with a resonance frequency of 30–40 KHz and a radius of 20 nm. Before analyzing them, the liposomes were sonicated. For each determination 0.2 mL was placed on a glass support and allowed to dry at a temperature of 25 °C for 60 min at a constant fan ventilation speed, and then at a temperature of 20 °C [22,23].

In order to evaluate the entrapment efficiency of CA (EE%), the unincorporated CA was removed from the nanovesicles. They were centrifuged, then various organic solvents (methanol, Triton X-100, ethanol) were used to destroy the vesicles. To extract the amount of incorporated CA, the destroyed vesicles were purified [27].

Studies analyzing the release of the substance from liposomes provide us data on the pharmacokinetics and bioavailability of CA. A Franz diffusion cell (Microette-Hanson system, model 57-6AS9, Copley Scientific Ltd., Nottingham, UK) was used, according to a previously published method [22,23]. This cell consists of a donor and acceptor compartment, separated by membranes of synthetic, human, or animal origin [28]. For this study, the synthetic membranes were used because these ensure a good simulation of the skin in the most realistic way with a good quality control.

### 2.2. Release Kinetics Analysis

Cumulative release was expressed as a percentage of the amount of encapsulated caffeic acid (considered 100%). For kinetic analysis, Higuchi, Korsmeyer–Peppas, First-order, and Zero-order models were applied using data from the initial release phase (first 5 h), corresponding to the ≤60% release range.

### 2.3. In Vitro Studies

#### 2.3.1. Reagents

Doxorubicin hydrochloride was purchased from Sigma-Aldrich (Merck KGaA; Darmstadt, Germany) and DMEM high glucose, 10% feral bovine serum, L-glutamine insulin, Non-Essential Amino Acids Solution, Penicillin-Streptomycin, and RPMI-1640 were purchased from Gibco (Termo Fisher Scientific, Waltham, MA, USA). Antibodies against nuclear factor erythroid 2-related factor (NRF)2, signal transducer and activator of transcription (STAT) 3, glyceraldehyde 3-phosphate dehydrogenase (GAPDH) and secondary peroxidase-linked antibodies were purchased from Santa Cruz Biotechnology (Delaware Ave, Santa Cruz, CA, USA), while phosphorylated histone H2AX (pS139) (γH2AX) was purchased from Stressgen Bioreagents Corporation (Victoria, BC, Canada). Bradford total protein concentration assay was purchased from Biorad (Hercules, CA, USA).

#### 2.3.2. Cell Cultures

Two lines of triple-negative breast cancer (TNBC) cells, parental Hs578T (HS) and Dox-resistant cells Hs578T (HSD), received by donation from Prof. Neagoe Berindan Ioana the UMF Functional Genomics Center, University of Medicine and Pharmacy Iuliu Hațieganu Cluj Napoca) and Olga Soritau from Ion Chiricuță Oncologic Institute Cluj Napoca, were used for testing the toxicity of Dox, CA, the two liposomes prepared with CA (DPPC, CNA) and two liposomes prepared without CA (eDPPC, eCNA). The Doxorubicin-resistant cell line was established by multiple dose exposure in 12 passages. The Dox dose which maintains the drug-resistance of Hs578T cells was 50 nM [19]. The two types of cell lines were kept in humidified incubator, at 37 °C with 95% air and 5% of CO_2_. Both cell lines were multiplied in DMEM high glucose supplemented with 10% feral bovine serum, 2 mM L-glutamine, 0.01 mg/mL insulin, 1% Non-Essential Amino Acids Solution (100X) and 1% Penicillin-Streptomycin, as previously described [29].

#### 2.3.3. Viability Assay

The Hs578T (HS) cells survival under exposure to CA, liposomes loaded with CA (DPPC, CNA) and liposomes without CA (eDPPC, eCNA) in presence or not to Dox, were tested through the colorimetric measurement of formazan using CellTiter 96^®^ AQueous Non-Radioactive Cell Proliferation Assay (Promega Corporation, Madison, WI, USA). The HS cells were cultivated at a density of 10^4^/well in 96 wells plaques (TPP, Trasadingen, Switzerland), for 24 h and then were treated for 24 h with the tested compounds (CA, DPPC, eDPPC, CNA, eCNA ranged between 10 and 160 µM) alone or under exposure to 50 µM Dox. The dose of Dox was chosen based on previous published experiments [19]. The optical density values were read at an absorbance of 540 nm using an ELISA plate reader (Tecan, Männedorf, Switzerland). Data were expressed as a mean of OD540 ± SD. All the experiments were conducted in triplicate independently. Untreated cultures, exposed only to medium, were used as controls. The toxic doses were considered those that decrease viability below 70% toxicity. Based on the viability results, in order to evaluate the cell death and the mechanisms involved, only CNA and eCNA liposomes were used, both on HS and HSD cells.

#### 2.3.4. Cell Lysates Preparation

For testing the effects of CA or liposomes loaded with CA (CNA) and without CA (eCNA) in HS and HSD cells exposed to Dox, the cells seeded on Petri dishes at a density of 10^4^/cm^2^, were exposed for 24 h to the tested compounds. To compare the effects of liposomes, CA alone and Dox alone, cells unexposed to Dox as well as untreated cells were used. CA, CNA and eCNA were used in a dose of 5 µM and treatments were performed concomitantly with exposure to 50 µM Dox. After 24 h, the cells were collected and the cell lysates were obtained using a standard procedure published before [30]. Protein levels in the samples were measured by using Bradford method (Biorad, Hercules, CA, USA) [31,32]. All the experiments were performed in triplicate.

#### 2.3.5. Apoptosis Evaluation

Both TNBC lines cultivated at a density of 10^4^/cm^2^ in Petri dishes were incubated for 24 h with 5 µM CA alone, 50 µM Dox alone or 50 µM/5 μM Dox/CA, 50 µM/5 μM Dox/CNA and 50 µM/5 μM Dox/eCNA. Unexposed cells were used as control. The apoptosis was detected by BD Annexin V-FITC/PI Assay (Franklin Lakes, NJ, USA). After treatment, the TNBC cells were stained with Annexin V-fluorescein isothiocyanate (FITC)/vital dye propidium iodide (PI) (BD Pharmingen Biosciences, San Jose, CA, USA) for 15 min, according to the previously published work [30,33]. All samples were analyzed with a BD FACS Canto II flow cytometer (Becton Dickinson & Company, Franklin Lakes, NJ, USA). Viable cells (Annexin V (-), PI (-), early apoptotic cells (Annexin V (+), PI (-), late apoptosis (Annexin V (+), PI (+) and necrotic cells (Annexin V (-), PI (+) were expressed as % of the total cell count.

#### 2.3.6. Oxidative Stress and LDH Activity Assessment

For quantification of the oxidative stress, the malondialdehyde (MDA was measured using the fluorometric method, according to the previously published work [34]. Lactate dehydrogenase (LDH) activity is a marker used to assess the cell membrane damage. LDH activity was quantified from the cell supernates using the spectrophotometric method as previously published [32] and the results was expressed in units/L.

#### 2.3.7. Evaluation of Transcription Factors and DNA Damage

The expression of transcription factors NRF2 and STAT3 and the DNA lesions quantified by γH2AX expression were assessed by western blot [33]. Thereby, lysates (20 mg protein/lane) obtained from TNBC cells were separated by electrophoresis on SDS PAGE gels and then transferred to polyvinylidene-difluoride membranes. Blots were blocked and then incubated with corresponding antibodies against NRF2, STAT3, γH2AX and GAPDH. GAPDH was used as the protein loading control. Proteins were distinguished using Supersignal West Femto Chemiluminescent substrate (Thermo Fisher Scientific, Waltham, MA, USA) and a Gel Doc Imaging system using Quantity One analysis software (Bio-Rad, v.4.6.1.).

### 2.4. Statistical Analysis

One-way ANOVA test was used, followed either by Tukey post hoc test to assess statistical significance between the groups for apoptosis rate, oxidative stress, membrane lesions and western blot analysis. Data were statistically processed using the GraphPad Prism software, version 10.6.1 (GraphPad Software, San Diego, CA, USA). All reported data were expressed as the mean of triplicate measurements ± SD and a *p* value lower than 0.05 was considered statistically significant (* vs control, untreated cells and ^#^ vs. Dox treatment).

## 3. Results

### 3.1. Preparation of Liposomes and Their Physico-Chemical Characterization

In order to obtain vesicle formulations, combinations of phospholipids (PC, DPPC) and cholesterol with a molar ratio of 10:1 were used. These phospholipids were chosen for liposomes preparation because the literature recommends them for obtaining colloidal particles for human use [35].

The phosphate buffer with pH = 7.6 was used to hydrate the lipid film for two reasons: liposomes form spontaneously at neutral pH, and phosphate salts help increase the stability of liposomes because they negatively charge their membrane [36]. The stability of liposomes is enhanced by adding the cholesterol fraction.

For characterization of liposomes, the particle size and zeta potential were performed on CNA and eCNA comparative with DPPC and eDPPC. Additionally, atomic force microscopy measurements, the entrapment efficiency of CA (EE%) and in vitro release of CA entrapped in liposomes were assessed. The particle size and zeta potential are shown in Table 2 and classification of samples after nanoparticles size is in Table 3. The zeta potential values suggest a high stability of CNA liposomes comparative with DPPC or eCNA liposomes. This is the main reason why they were chosen for evaluating the effects in biology.

### 3.2. Atomic Force Microscopy Measurements

The analysis of liposomes loaded with CA was done by comparison with the corresponding liposomes without CA (Table 4).

The AFM values from eCNA and eDPPC are identical to those published in a previous article [22], as they are determinations made on the empty liposomal matrix.

**Figure 1:**
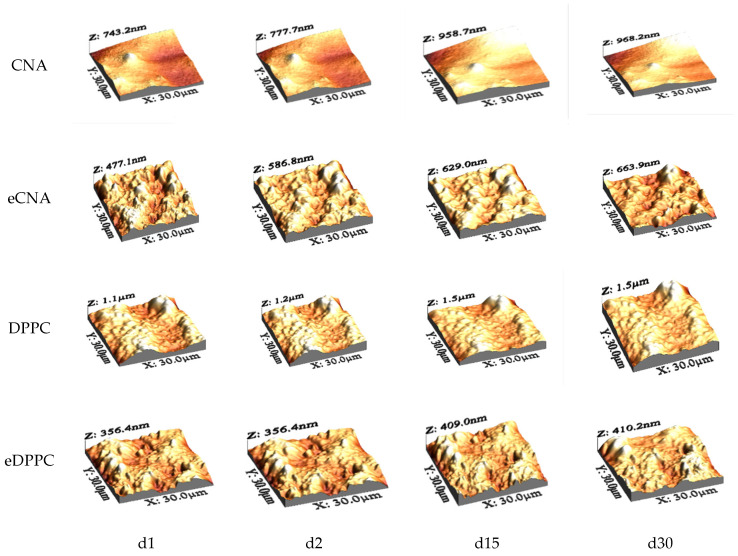
3D images obtained at AFM for CNA, DPPC, eCNA and eDPPC liposomes at different time intervals.

The eCNA, eDPPC samples show an upward trend for the parameters Sa, Sq, Sp, Sv and Sy. To assess the stability over time of the synthesized liposomes, 3D determinations were performed at the following time intervals: day 1 (d1), day 2 (d2), day 15 (d15), and day 30 (d30). The 3D images of the CNA, DPPC, and eCNA, eDPPC liposomes obtained at AFM can be found in Figure 1. 3D images for eCNA and eDPPC were published in a previous article [22].

AFM analysis qualitatively confirmed the presence of nanoscale vesicle-like structures in the liposomal formulations (Figure 1). As the images presented in Figure 1 are limited in terms of individual vesicle visualization, we have included additional AFM data in the Supplementary Material (Figures S1–S4), which include height profiles plotted over representative vesicles, as well as the evolution of histograms over time obtained from roughness data, allowing a clearer appreciation of the surface morphology and topography. Given that AFM imaging of soft, hydrated vesicles deposited on a solid support is subject to the risk of tip-induced deformation and lateral broadening, these results are interpreted predominantly qualitatively and used as a complementary confirmation of the presence of liposome-like structures, while the quantitative size distribution is mainly derived from DLS measurements.

The size observed by AFM was slightly lower than the hydrodynamic diameter obtained by DLS, which can be attributed to sample dehydration during AFM preparation.

The entrapment efficiency of CA (EE%) of CNA liposome is 86.87 ± 1.12, and for DPPC is 85.24 ± 0.99. The amount of CA released from the two types of liposomes was determined using a UV-VIS spectrophotometric method, the reading being performed at 325 nm. Three determinations were performed, and the results are presented (Table 5) as mean ± standard deviation.

The values for CA have been previously published [22]; however, for ease of comparison, they are also provided here. It was noticed that the release of CA from liposomes was time-dependent and the amount of CA trapped in CNA liposomes increased up to 3 h and remained elevated at the same level for 48 h (Table 5), demonstrating their stability over time.

Based on their stability, high entrapment capacity and a gradual release profile of CA as well as the results obtained in the cell viability assay, we set out to continue studies to evaluate the biological activity of CNA and eCNA liposomes compared to CA, Dox and Dox/CA in two cell lines exposed to Dox compared to CA or Dox alone.

To elucidate the mechanism of caffeic acid release from CNA and DPPC liposomes, the experimental data were fitted according to the Zero-order, Higuchi, Korsmeyer–Peppas and First-order kinetic models.

The models were applied using data from the initial release phase (0.5–5 h), corresponding to a cumulative release percentage ≤60%, an interval in which the mathematical assumptions of the models are applicable. The kinetic parameters obtained by fitting the Zero-order, Higuchi, Korsmeyer–Peppas and First-order methods are presented in Table 6.

All models showed high correlation coefficients (R^2^ > 0.97), suggesting that the release of caffeic acid from both liposomal systems is governed by a complex mechanism.

The Higuchi model supports the contribution of the diffusion process in the initial stage, while the adjustment according to the First-order model indicates a partial dependence of the release rate on the amount of substance remaining in the system (liposome).

According to the Korsmeyer–Peppas model, the release exponent values were *n* = 1.07 for CNA and *n* = 0.94 for DPPC, indicating an abnormal (non-Fickian) transport. The superunit value observed for CNA may suggest a more pronounced contribution of the relaxation or structural reorganization phenomena of the lipid layer in the initial phase of release.

### 3.3. Biological Activity

#### 3.3.1. Cell Viability Assay

**Figure 2:**
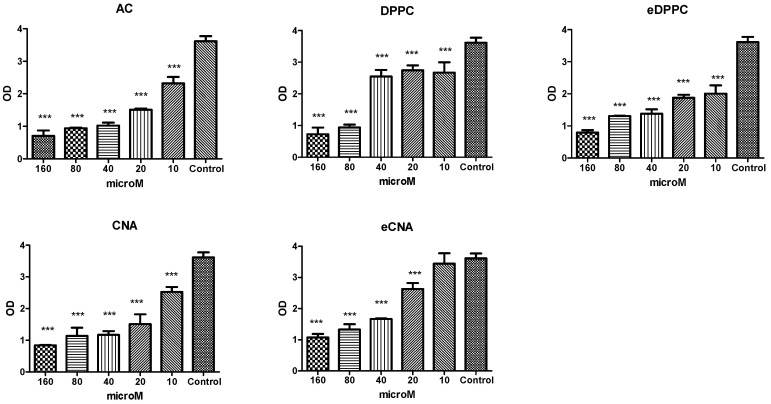
Cell viability of HS cells treated with CA, DPPC, eDPPC, CNA and eCNA liposomes. The cells were exposed to 50 μM Dox and treated with CA, DPPC, eDPPC, CAN and eCNA liposomes in concentrations ranging between 10 and 160 µM. Data are presented as a mean of OD540 ± SD; *n* = 3 for each sample. *** *p* < 0.001 vs. control, untreated cells.

**Figure 3:**
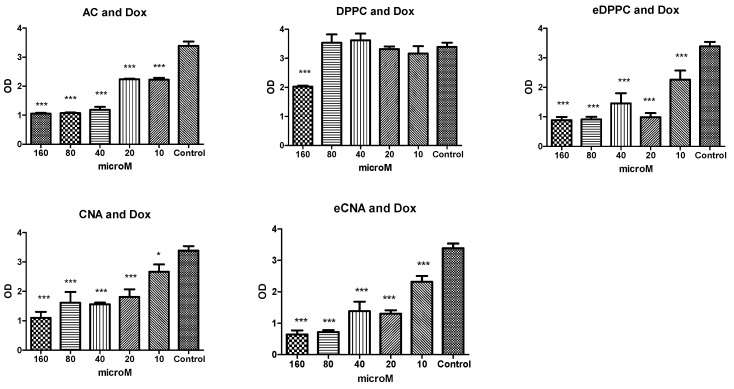
Cell viability of HS cells exposed to Dox and treated with CA, DPPC, eDPPC, CAN and eCNA liposomes. The cells were exposed to 50 μM Dox and treated with CA, DPPC, eDPPC, CAN and eCNA liposomes in concentrations ranging between 10 and 160 µM. Data are presented as a mean of OD540 ± SD; *n* = 3 for each sample. * *p* < 0.05, *** *p* < 0.001 vs. control, untreated cells.

To study the impact of CA alone and Dox alone or CA, CNA and eCNA in cells unexposed to Dox or in cells exposed to Dox, the MTS assay was performed. The cell viability was measured after exposure to different concentrations of all tested compounds alone (ranging between 10 μM and 160 μM) firstly without Dox exposure (Figure 2). CA decreased the cell viability at doses above 10 µM, while both formulations of liposomes entrapped with CA decreased the cellular viability at doses over than 10 µM, especially the CNA formulation (Figure 2). The eDPPC liposomes decreased the cell viability more than DPPC, while in the case of CNA liposomes, after entrapment with CA, the cell viability was seriously affected at concentrations above 10 μM. The decline of viability was dose-dependent for CA and the CNA liposomes formulation. In cells exposed to Dox and incubated with CA, or CNA and eCNA formulations, the pattern was different (Figure 3). Thus, DPPC liposomes maintained a good cell viability at all concentrations tested up to 80 μM, while the same liposome formula but without CA decreased the cell viability, suggesting its toxicity at concentrations above 10 μM. In these liposomes, CA preserved the cell viability up to 40 μM, while in the CNA, the viability decreased at values above 10 μM. The different behavior of the two formulations of liposomes depends on their composition. Based on the results at the viability assay, the liposomes used for other biology testing were CNA and eCNA, and the concentrations chosen for further experiments were 5 μM for all tested compounds.

#### 3.3.2. The Evaluation of Cell Apoptosis by FACS Analysis

**Figure 4:**
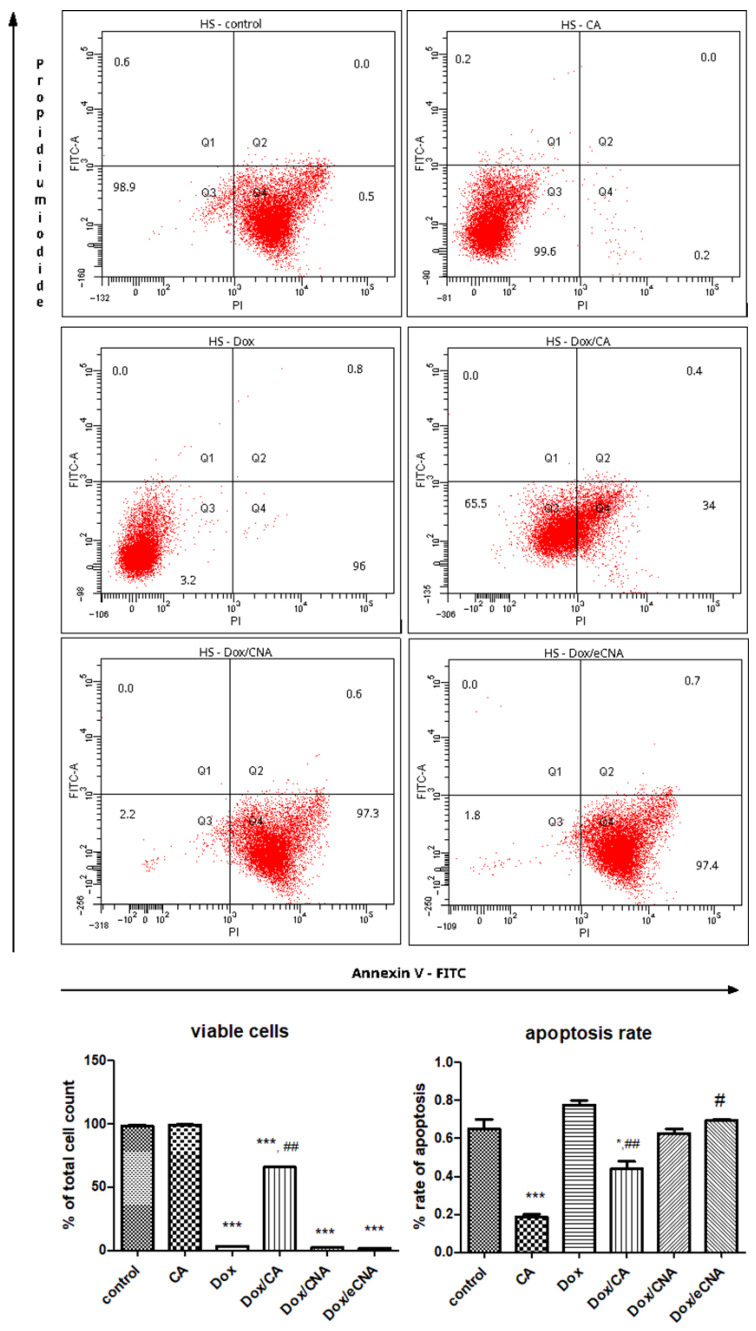
Flow cytometric analysis of HS cells treated with CA, Dox, Dox/CA, Dox/CAN and Dox/eCNA. The quantitative FACS results are expressed as % of HS cells from total cell count (upper panel). In the lower left panel, the graphs show the viable cells—Annexin V (-), PI (-), and in the lower right panel the non-viable cells, specifically the apoptotic cells (early apoptotic cells—Annexin V (+), PI (-), late apoptosis—Annexin V (+), PI (+) and necrotic cells (Annexin V (-), PI (+). Each bar represents means ± SD. CA maintained the same proportion of HS viable cells close to control, while Dox exposure, Dox/CNA or Dox/eCNA treatments reduced significantly (*p* < 0.001) the % of HS cells. The apoptotic rate decreased significantly after CA administration (*p* < 0.001) or Dox/CA treatment (*p* < 0.05) compared to control. The statistical significance of the difference between treated and control groups was evaluated with one-way ANOVA followed by Tukey posttest. * *p* < 0.05, *** *p* < 0.001 vs. control; ^#^ *p* < 0.05 and ^##^ *p* < 0.01 vs. Dox exposure.

**Figure 5:**
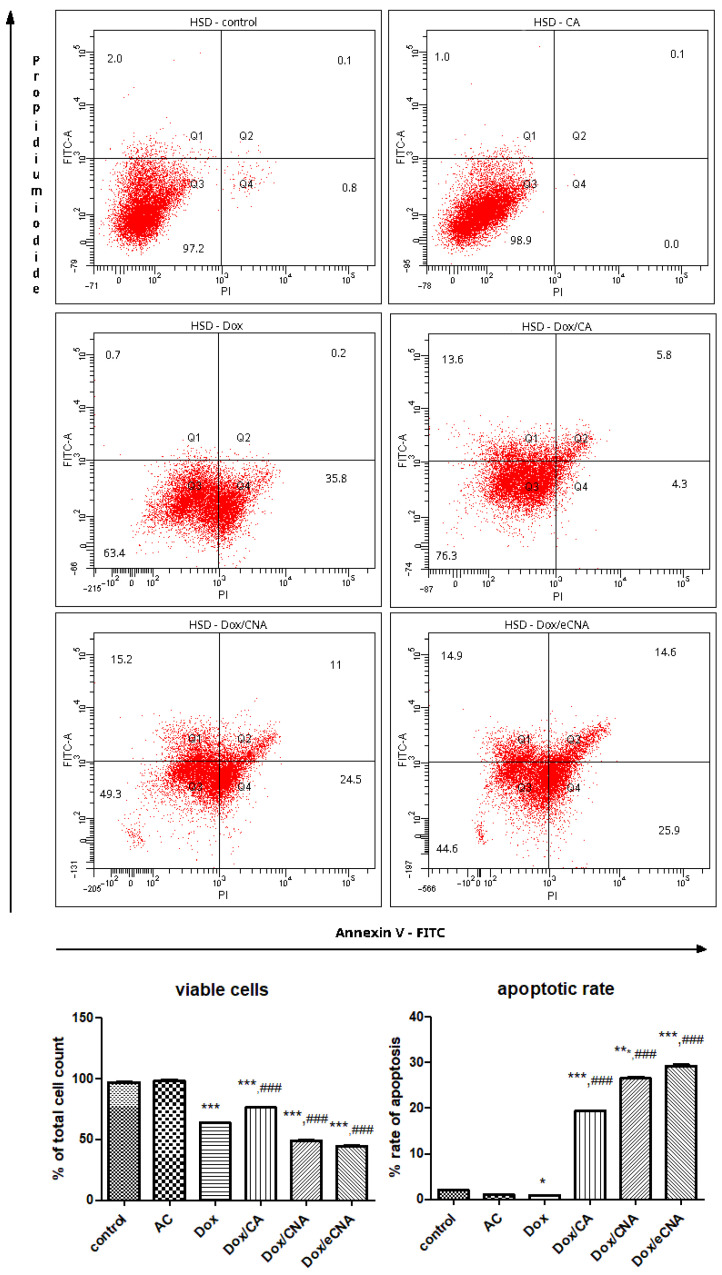
Flow cytometric analysis of HSD cells treated with CA, Dox, Dox/CA, Dox/CAN and Dox/eCNA. Dox diminished the percentage of viable cells compared to control (*p* < 0.001), but the cell death by apoptosis was insignificant. Dox/CNA and Dox/eCNA reduced the % of viable cells compared to Dox (*p* < 0.001) or control (*p* < 0.001) and increased the apoptotic rate. The same effects were obtained after Dox/CA administration compared to Dox exposure (*p* < 0.001). The quantitative FACS results are expressed as % of HSD cells from total cell count (upper panel), while in the lower left panel, the graphs show the viable cells—Annexin V (-), PI (-). In the lower right panel are presented the non-viable cells, specifically the apoptotic cells (early apoptotic cells—Annexin V (+), PI (-), late apoptosis—Annexin V (+), PI (+) and necrotic cells (Annexin V (-), PI (+). Each bar represents means ± SD. The statistical significance of the difference between treated and control groups was evaluated with one-way ANOVA followed by Tukey posttest. * *p* < 0.05 and *** *p* < 0.001 vs. control; ^###^ *p* < 0.001 vs. Dox exposure.

The mechanism of cell death assessed by Annexin V/propidium iodide staining showed that the administration of CA maintained the same proportion of HS viable cells close to control (Figure 4). The % of viable cells decreased after Dox exposure (*p* < 0.001) or Dox/CAN and Dox/eCNA treatments (*p* < 0.001) of HS cells. The apoptotic rate diminished significantly after CA administration (*p* < 0.001) or Dox/CA treatment (*p* < 0.05) compared to control. Dox exposure did not influence the apoptosis of HSD cells, while eCNA formula increased the cell death (*p* < 0.05) (Figure 5).

In HSD cells, Dox diminished the percentage of viable cells compared to control (*p* < 0.001), but the cell death by apoptosis was insignificant. Dox/CNA and Dox/eCNA reduced the % of viable cells compared to Dox (*p* < 0.001) or control (*p* < 0.001), while Dox/CA slightly increased the cell viability compared to Dox treatment. The apoptotic rate of HSD cells was significant after Dox/CA, Dox/CNA or Dox/eCNA treatments suggesting the beneficial of CA or liposomes in association with Dox in the occurrence of cell death (*p* < 0.001).

#### 3.3.3. Oxidative Stress and LDH Activity

**Figure 6:**
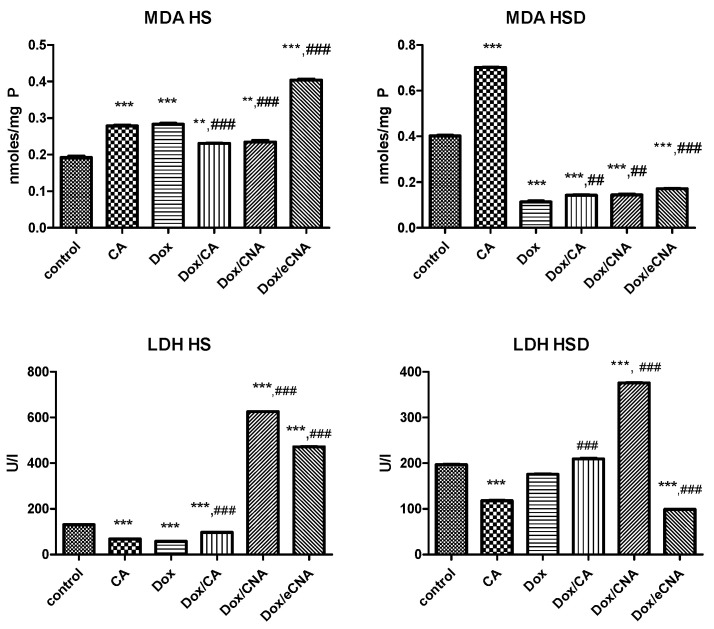
MDA and LDH levels in cell lysates of cells exposed to Dox and treated with CA, CNA and eCNA liposomes. The HS and HSD cells were exposed to Dox and treated with CA, CNA and eCNA liposomes and the results were compared to CA alone and Dox alone. MDA levels increased significantly after treatment of HS cells with CA or Dox alone compared to control (*p* < 0.001). The MDA values decreased after incubation with the CNA formula or CA in HS cells exposed to Dox, while the eCNA formula significantly increased the lipid peroxidation (*p* < 0.001) compared to the control or Dox treatment. In HSD cells, CA enhanced significantly MDA levels (*p* < 0.001), while all tested compounds maintained low levels of MDA compared to control (*p* < 0.001). LDH activity diminished after CA or Dox (*p* < 0.001) administrations and increased significantly in HS cells treated with CNA and eCNA (*p* < 0.001). In HSD cells, LDH activity decreased in supernates of cells treated with CA, while after Dox, Dox/CA and Dox/CNA increased (*p* < 0.001). The statistical significance of the difference between treated and control groups was evaluated with one-way ANOVA, followed by the Dunnett multiple test. Data are presented as mean and SD of triplicates samples. ** *p* < 0.01, *** *p* < 0.001 vs. control; ^##^ *p* < 0.01, ^###^ *p* < 0.001 vs. Dox treated cells.

MDA levels increased significantly after treatment of HS cells with CA or Dox alone compared to control (*p* < 0.001) (Figure 6). The MDA values decreased after incubation with CAN formula or CA in HS cells exposed to Dox, while eCNA formula increased significantly the lipid peroxidation (*p* < 0.001). In HSD cells, CA treatment enhanced significantly MDA levels (*p* < 0.001) compared to control, while all tested compounds maintained low levels of MDA in cells exposed to Dox. Moreover, Dox reduced MDA levels in HSD cells suggesting the resistance to oxidative stress of these cells.

The cell membrane integrity was evaluated by LDH activity measurements in supernates (Figure 6). The enzyme activity increased in HS cells treated with CA or Dox compared to control (*p* < 0.0010 and decreased after Dox/CA or Dox/CNA administration (*p* < 0.001). The highest damage of cell membrane was noticed after Dox/eCNA exposure, both compared to control or compared to Dox alone (*p* < 0.001). In HSD cells, the LDH activity increased significantly after CA treatment (*p* < 0.001) and was reduced in all treatments performed (Dox, Dox/CA, Dox/CNA or Dox/eCNA) (*p* < 0.001). The lowest activity of LDH was noticed after exposure of HSH to Dox.

#### 3.3.4. Evaluation of Transcription Factors and DNA Lesions

**Figure 7:**
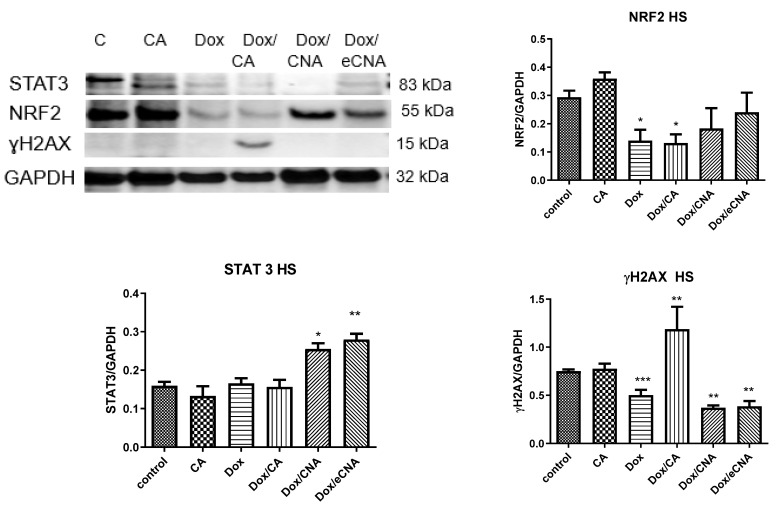
NRF2, STAT3 and ɣH_2_AX expressions in HS cells treated with CA, Dox, Dox/CA, Dox/CNA and Dox/eCNA. Image analysis of WB bands was done by densitometry (upper panel); results were normalized to GAPDH (lower panel) (STAT3 86kDa; NRF2 55 kDa; ɣH_2_AX 15kDa and GAPDH 35 kDa). The statistical significance between treated cells and control group was assessed with the one-way ANOVA followed by the Tukey posttest. Each bar represents mean ± standard deviation (n = 3); * *p* < 0.05, ** *p* < 0.01, *** *p* < 0.001 vs. control, untreated cells.

**Figure 8:**
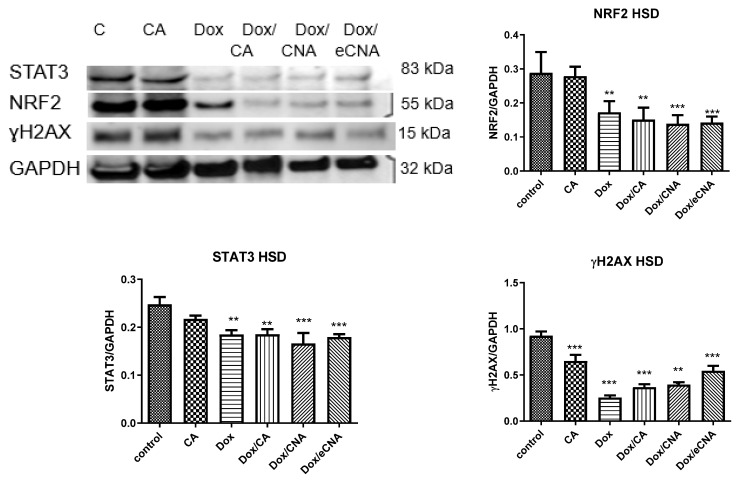
NRF2, STAT3 and ɣH_2_AX expressions in HSD cells treated with CA, Dox, Dox/CA, Dox/CNA and Dox/eCNA. Image analysis of WB bands was done by densitometry (upper panel); results were normalized to GAPDH (lower panel) (STAT3 86kDa; NRF2 55 kDa; ɣH_2_AX 15kDa and GAPDH 35 kDa). The statistical significance between treated cells and control group was assessed with the one-way ANOVA followed by the Tukey posttest. Each bar represents mean ± standard deviation (n = 3); ** *p* < 0.01, *** *p* < 0.001 vs. control, untreated cells.

In our experimental design, CA treatment of HS cells induced significantly NRF2 expression without influence the STAT 3 expression (*p* > 0.05) (Figure 7). Dox alone reduced DNA lesions of HS cells compared to control, while the combination Dox with CA (*p* < 0.001) increased ɣH_2_AX foci formation (*p* < 0.01). DNA lesions induced by Dox/eCNA were close to Dox but STAT3 expression increased significantly (*p* < 0.001). The same effect on STAT 3 expression was found after Dox/CNA treatment (*p* < 0.01). In HSD cells NRF2, STAT3 and ɣH_2_AX expressions decreased significantly after Dox (*p* < 0.01), Dox/CA (*p* < 0.01), Dox/CNA (*p* < 0.001) and Dox/eCNA (*p* < 0.001) (Figure 8). The same pattern was noticed after CA administration (*p* < 0.01)

## 4. Discussion

The triple-negative breast cancer is considered a challenge for oncologists due to a lack of response to endocrine or targeted therapies and is associated with a high metastatic potential and an unfavorable prognostic rate of the tumor. TNBC occurs frequent in young people and the effectiveness of chemotherapy or radiotherapy is often negatively marked by increased resistance to treatment [19]. Doxorubicin is a common chemotherapeutic agent used in treatment of breast cancer in combined schema with paclitaxel or/and cyclophosphamide [10,11,12] but sometimes chemoresistance occurs due to activation of pro-survival signaling, alteration of DNA repair mechanisms and epithelial–mesenchymal transition [14,17]. Therefore, the finding of additive compounds with selective cytotoxicity on cancer cells, high stability and penetrability to the target site, can be an option in therapeutic strategy of aggressive tumors.

It is known that TNBC cell lines possess intrinsic marked resistance to oxidative stress and genomic instability with high γ-H2AX foci formation and constitutive activation of the STAT3 pathway [19,21]. These cells were chosen to evaluate their response to liposomes loaded with CA in association with Dox compared to CA, Dox, or liposomes free of CA. In our study, CA was selected because of its significant antitumoral properties demonstrated in previous studies. Thus, it was demonstrated that CA and its derivative CADPE inhibited STAT3 activity, HIF-1a and VEGF expressions, prevented the tumor growth, and controlled the excessive angiogenesis in tumor cells [5,6]. The mechanisms involved the suppression of STAT3 phosphorylation at tyrosine-705 and inhibition of STAT3 translocation to the nucleus [37]. Additionally, CA inhibited TNBC cell proliferation, suppressed oxidative stress, modulated the FOXO1/FIS signaling pathway, induced mitochondrial autophagy, and exerted an antitumor effect by amplifying the autoimmune response of macrophages [38]. Caffeic acid phenethyl ester (CAPE) administration reversed Dox resistance in breast cancer cells due to inhibition of protein kinase B (Akt)/mammalian target of rapamycin (mTOR)/sterol regulatory element binding protein 1 (SREBP1) pathway and lipid metabolism [39]. In our experiment, an important entrapment of CA in liposomes was noticed probably due to low solubility of CA in water at room temperature [22] and gradual release of CA from liposomes. A similar encapsulation efficiency was obtained by Katuwavila et al. [40] using egg phosphatidylcholine liposomes or by Zaremba-Czogalla et al. which prepared liposomes using soybean phosphatidylcholine and DSPE-PEG2000 (Alabaster, AL, USA) [41]. Although the Zero-order model provided the highest values of the correlation coefficient, the simultaneous agreement with the Higuchi and First-order models indicates that the release does not follow strictly constant kinetics, but results from the superposition of several physico-chemical processes.

The similarity of the Higuchi constants suggests that diffusion through the phospholipid bilayer represents a dominant mechanism in both formulations. However, the differences observed at the level of the exponent “n” highlight variations in the membrane dynamics. In the case of CNA, the value n > 1 suggests a more pronounced contribution of the phenomena of relaxation or reorganization of the lipid structure, possibly due to a different degree of packing or fluidity of the membrane.

These results support the hypothesis that the lipid composition influences not only the release rate, but also the dominant mechanism in the initial phase, highlighting the role of the structural properties of the bilayer in controlling the release of the active substance.

Overall, kinetic modeling confirms the complex nature of release from liposomal systems and reveals the importance of lipid composition in modulating early release behavior.

In our research, CA administration maintained the proportion of viable cells due to reduction in apoptotic cell death in HS cells and increased lipid peroxidation, while in HSD cells, CA treatment did not influence the cell viability and apoptosis, but increased the MDA levels. In HS cells exposed to Dox, CA treatment improved the number of viable cells, while in HSD cells it enhanced the apoptosis as a mechanism of cell death and decreased slightly but insignificantly STAT3 expression. In HS cells, Dox treatment associated with CA administration triggered statistically significant DNA lesions compared to Dox. Liposomes loaded with CA exerted apoptotic effects on both cells, comparable with Dox, and increased the cell membrane lesions. Additionally, CNA liposomes increased STAT3 in HS cells, while in HSD cells, they diminished NRF2 and STAT3 expressions, suggesting beneficial effects on the breast tumor cells resistant to Dox.

It is known that STAT3 is activated in human cancer and consequently represents an important target in developing new anticancer therapies. STAT3 directly induces the most angiogenic molecule [37], specifically vascular endothelial growth factor (VEGF) and upregulates the transcription of genes involved in cell survival and cell proliferation, in anti-apoptosis and immune response [38]. In our study, the inhibition of STAT3 was significant in HSD cells, especially in cells exposed to Dox and treated with liposomes entrapped with CA, showing the promise of liposomes loaded with CA in reducing tumor growth and in reversing chemoresistance of resistant tumor cells.

Some experimental findings demonstrated the inhibitory properties of caffeine and CA on human breast cancer cell proliferation in MCF-7, T47D (both ERα-positive), and MDA-MB-231 (ERα^−^) cell lines [40]. Moreover, caffeine and CA suppressed the growth of estrogen receptor (ER)+ and ER- cells and reduced cyclin D1 levels in cells positive for ER and insulin-like growth factor-I receptor (IGFIR). In ER+ and ER- cells, CA diminished pAKT levels, suggesting inhibition of survival pathways and important antiproliferative effects [39,42].

Jung et al. (2007), highlighted that CA inhibited tumor angiogenesis in renal carcinoma cells by blocking the activation of STAT3-mediated activation of vascular endothelial growth factor (VEGF) and hypoxia-inducible factor 1α (HIF-1α) [37]. In our study, in both tumor cells, without exposure to Dox, CA treatment maintained the cell viability and cell death close to the control, untreated cells, and protected the tumor cells by increasing the antioxidant defense adaptively, in response to redox imbalance, especially in HS cells. In presence of Dox, CA induced apoptosis and inhibited STAT3 expression of HSD cells in parallel with the increase in membrane damage. In HS cells treated with Dox, CA administration protected the tumor cells and triggered DNA damage, but at an insufficient level to promote cell death.

Liposomes loaded with CA exerted antitumor effects, reduced the viable cells and induced apoptotic cells death and also increased cell membrane damage in both tumor cells, compared to Dox. The mechanisms of cell death were different in the two types of cell lines. Thus, in HS cells exposed to Dox, CNA liposomes induced cell membrane damage, while in HSD cells, they reduced the antioxidant defense and generated important membrane cell lesions in the presence of Dox, in parallel with downregulation of pro-survival transcription factors, STAT3 and NRF2 without to influence DNA. Unlike Doxorubicin, which directly attacks DNA, an effect demonstrated especially in HS cells, liposomes target cellular communication and protection pathways, providing a complementary method of attack to Dox. Thus, liposomes reduce STAT3 and NRF2 expression and induce cell membrane damage, beneficial effects in reducing tumor cell survival. Moreover, liposomes, being lipid structures, could better protect against caffeic acid degradation and release targeted into the acidic tumor environment, and after fusion with the cell membrane, could damage it more easily.

It is known that apoptosis is a form of programmed cell death that occurs for old, damaged, or disrupted cells and represents a crucial process for normal cell turnover, tissue homeostasis, and cancer prevention [43]. This process can be initiated through two distinct mechanisms, extrinsic and intrinsic, and involve activation of caspases in signaling cascades as well as proapoptotic and antiapoptotic proteins and finally leads to cell shrinkage, chromatin condensation, formation of apoptotic bodies which will be phagocytized by adjacent parenchymal cells, neoplastic cells or macrophages [44].

Flowcytometric analysis of HS cells showed that CA maintained the same proportion of viable cells close to control, while Dox exposure, associated or not with CNA or eCNA administration, significantly reduced cell viability. HSD cells are more resistant to apoptosis, and only Dox/CA or liposomes loaded or not with CA can induce cell apoptosis. Several studies showed that Dox induced apoptosis by decreasing NF-κB and anti-apoptotic Bcl-2 protein expressions and by upregulating Bax, caspase-8, and caspase-3 [45]. Overproduction of ROS, mainly formed in mitochondria, can trigger mitochondrial membrane permeability and release of cytochrome C, and consequently, apoptotic death occurs [46]. Redox imbalance also activate transcription factors such as NRF2, NF-ĸB, and activator protein 1 (AP-1) [47] and stimulate mitogen-activated protein kinase (MAPK) signal transduction cascades [48]. Moreover, overproduction of ROS occurs in cancer cells and alters proteins, DNA, and lipids from cells, inducing cell membrane lesions [49], activation of inflammasomes, and the initiation of pyroptosis and apoptosis. Therefore, the ROS generation and cell membrane lesions occurrence and induction of apoptosis may be effective strategy in triple-negative breast cancer treatment. Liposomes loaded with CA associated with Dox reduced the cell viability, enhanced the apoptotic rate, and triggered cell membrane damage in breast cancer-resistant cells, suggesting the potential of this compound for the treatment of aggressive tumors.

It is known that NRF2 is a transcription factor that protects the cells from oxidative stress and inflammation-induced cellular damage, and, in tumor cell lines, NRF2 increases the resistance of cells to chemotherapy and radiotherapy [50,51,52]. NRF2 ensures the cytoprotection through NADPH important regulator of several enzymes involved in glucose metabolism and ferritin production [53,54,55]. In tumor cells, NRF2 favors the carcinogenesis by inhibiting of apoptosis and inducing angiogenesis by heme oxygenase 1 (HO-1), hypoxia-inducible factor 1 (HIF-1) [56], and VEGF A (VEGFA) expressions [55]. In HSD cells treated with CNA liposomes, the NRF2 expression decreased in correlation with an increased rate of apoptosis, suggesting a harmful effect on the survival and protection mechanisms developed in tumor cells. It seems that oxidative stress plays a significant role in cellular functions regulated by NRF2. NRF2 is a transcription factor required for cellular homeostasis: it enhances the activity of cytoprotective genes such as glutamate cysteine ligase (GCS) and quinone oxidoreductase-1 (NQO1), thus protecting against oxidative stress. The chemopreventive effect of some agents is due to the activation of NRF2 by preventing the protein degradation or promoting NRF2 gene transcription [57], a process that takes place in the presence of reactive oxygen species. Chemotherapy resistance is caused by the activation of NRF2 in cancer cells [58] and its inhibition by different drugs and siRNA administration or by inhibition of NADPH quinone oxidoreductase can improve the therapeutic effect in breast cancer tissues and breast tumor cells (MDA-MB-453, MCF-7, MDA-MB-231, MDA-MB-468) [59]. NRF2 inhibition in HSD cells by Dox associated with liposomes loaded with CA or without CA confirmed the efficacy of the tested compounds in overcoming the resistance of these cells.

γH2AX foci formation is a marker of DNA double-strand breaks (DSBs) and signifies the overcoming of DNA repair mechanisms, reflecting high resistance to radiation or chemotherapy. Triple-negative breast cancer cell lines often possess high baseline or induced γ-H2AX levels, and, therefore, the identification of compounds that alone or in combination with chemotherapy or radiotherapy can overcome this target is an important strategy in resistant cells treatment. In our study, Dox/CA caused DNA damage compared to Dox or CA in HS cells, while in HSD cells their effect on γ-H2AX formation was significantly reduced.

## 5. Conclusions

Finding new agents with selective cytotoxicity on cancer cells, with minimal toxicity on normal tissues, represents an important strategy in oncology. CA was chosen for testing due to its known antiproliferative ability, and the formulation in liposomes can offer higher stability and increased efficacy on tumor cells. Triple-negative tumor cells are aggressive due to intrinsic resistance mechanisms, and overcoming their survival mechanisms is a real therapeutic challenge in oncology.

CNA liposomes entrapped with CA possess a great stability and a time-dependent release of CA. They induced apoptotic cell death and cell membrane lesions and diminished the expression of survival molecules in tumor resistant cells suggesting beneficial properties on their therapeutic management. Only CA administration maintained the proportion of viable cells due to a reduction in apoptotic cell death in HS cells and increased lipid peroxidation, but this was insufficient to cause cell death, while in HSD cells, CA treatment did not influence the cell viability and apoptosis, the values being close to those of untreated cells. Although CA induced lipid peroxidation in HSD, this mechanism is not sufficient to reduce tumor cell viability in resistant tumor cells. In cells exposed to Dox, CA improved slightly the number of HS viable tumor cells but at the same time increased the apoptotic rate, while in HSD cells it enhanced the apoptosis as a mechanism of cell death and decreased the expression of survival molecules. With all these promising results, some limitations in the present study should be considered. HS and HSD cells are tumor cells resistant to the inhibitory agents previously effective on other tumor types, suggesting that the results cannot be generalized to all forms of cancer. The combination between CA and Dox showed promising results in HSD cells by potentiating apoptosis, suggesting a potential role of CA as an adjuvant in overcoming chemoresistance. Moreover, CA has limited effectiveness and modest impact in combination with chemotherapy which justifies the need to test nano-structured transport systems to overcome tumor defense mechanisms. Although they have demonstrated beneficial effects on apoptosis, membrane lesions and on transcription of survival molecules, further studies are necessary to quantify other survival pathways expressed by resistant cells. Probably, it would have been useful to evaluate various doses of CA or different exposure times in order to determine the optimal threshold at which free CA could switch from a protective (antioxidant) to a cytotoxic (pro-oxidant) effect in resistant cells.

In conclusion, these results indicated that liposomes entrapped with CA were able to interfere with some mechanisms of triple-negative cell survival and inhibit their proliferation.

## Figures and Tables

**Table 1:** Quantities of substances used for liposome synthesis.

Type of Liposome	The Amount of CA (mg)	The Amount of DP-PC (mg)	The Amount of PC (mg)	The Amount of CHL (mg)	The Amount of SC (mg)
DPPC	50	50	50	2.5	-
eDPPC	-	50	50	2.5	-
CNA	50	-	80	2.5	20
eCNA	-	-	80	2.5	20

**Legend:** CA—caffeic acid, DPPC and CNA loaded liposomes with CA; eDPPC and eCNA empty liposomes, CHL—cholesterol, SC—sodium cholate, PC—phosphatidylcholine, DP-PC—1,2-dipalmitoyl-sn-glycero-3-phosphocholine.

**Table 2:** Zeta potential for four liposomes.

Liposome Series	Liposome	Zeta Potential (mV)
With CA	DPPC	−0.271
	CNA	−7.66
Without CA	eDPPC	0.565
	eCNA	−10.9

**Table 3:** Classification of samples from series II of liposomes (with 50 mg CA entrapped) by nanoparticle size or nano-levels. Nano-levels: (1) 1–150 nm, (2) 150–500 nm and (3) 500–6000 nm.

Time (Day)	DPPC	CNA	Color Legend for Hierarchical Classification of Nano-Levels
**d1**	bimodal nano-levels (1) and (3)	unimodal nano-level (1)	nano-level (1)
**d15**	unimodal nano-level (1)	unimodal nano-level (1)	nano-levels (1) and (2) dominant and (3)
**d30**	trimodal nano-level (1)	bimodal nano-level (1)	nano-levels (1) dominant and (3)
			**•** nano-level (3)

**Table 4:** Evolution of AFM values for CNA, DPPC, eCNA and eDPPC samples.

Sample	Day	Ironed Area (µm^2^)	Sa (µm)	Sq (µm)	Sp (µm)	Sv (µm)	Sy (µm)
CNA	d1	916.32 ± 0.11	0.10 ± 0.00	0.12 ± 0.01	0.45 ± 0.01	−0.29 ± 0.01	0.74 ± 0.00
	d2	912.34 ± 0.11	0.10 ± 0.01	0.12 ± 0.00	0.46 ± 0.01	−0.32 ± 0.00	0.78 ± 0.01
	d15	913.94 ± 0.10	0.13 ± 0.01	0.16 ± 0.01	0.43 ± 0.00	−0.53 ± 0.00	0.96 ± 0.00
	d30	920.28 ± 0.10	0.13 ± 0.00	0.16 ± 0.01	0.48 ± 0.00	−0.49 ± 0.01	0.97 ± 0.00
eCNA	d1	904.49 ± 0.10	0.06 ± 0.00	0.07 ± 0.01	0.22 ± 0.00	−0.26 ± 0.00	0.48 ± 0.00
	d2	903.35 ± 0.10	0.06 ± 0.01	0.08 ± 0.01	0.25 ± 0.01	−0.33 ± 0.00	0.59 ± 0.01
	d15	902.62 ± 0.10	0.06 ± 0.00	0.08 ± 0.01	0.25 ± 0.00	−0.38 ± 0.00	0.63 ± 0.00
	d30	903.60 ± 0.10	0.07 ± 0.00	0.09 ± 0.00	0.35 ± 0.00	−0.31 ± 0.01	0.66 ± 0.00
DPPC	d1	907.25 ± 0.10	0.15 ± 0.01	0.18 ± 0.00	0.51 ± 0.00	−0.64 ± 0.00	1.1432 ± 0.01
	d2	906.98 ± 0.10	0.14 ± 0.00	0.17 ± 0.00	0.48 ± 0.01	−0.70 ± 0.00	1.18 ± 0.01
	d15	907.47 ± 0.10	0.15 ± 0.00	0.18 ± 0.00	0.64 ± 0.00	−0.81 ± 0.00	1.45 ± 0.00
	d30	907.32 ± 0.11	0.14 ± 0.00	0.18 ± 0.00	0.63 ± 0.01	−0.83 ± 0.01	1.45 ± 0.01
eDPPC	d1	901.39 ± 0.10	0.04 ± 0.00	0.06 ± 0.00	0.18 ± 0.00	−0.18 ± 0.01	0.36 ± 0.01
	d2	901.39 ± 0.10	0.04 ± 0.00	0.058 ± 0.00	0.18 ± 0.00	−0.18 ± 0.00	0.36 ± 0.01
	d15	902.12 ± 0.11	0.05 ± 0.00	0.21 ± 0.01	0.19 ± 0.01	−0.22 ± 0.01	0.41 ± 0.01
	d30	902.14 ± 0.11	0.05 ± 0.00	0.30 ± 0.01	0.18 ± 0.00	−0.23 ± 0.01	0.41 ± 0.00

**Table 5:** The percentage of CA released from CNA and DPPC liposomes synthesized as a function of time and CA amount trapped in CNA and DPPC liposomes.

Percentage of CA Released (%) from Liposomes	Time (h)											
	0.5	1	2	3	4	5	6	7	8	12	24	48
CNA	3.85 ± 0.00	12.43 ± 0.68	22.48 ± 1.09	33.49 ± 1.50	39.35 ± 3.01	51.99 ± 4.29	64.51 ± 3.55	77.41 ± 3.01	87.41 ± 4.62	88.80 ± 5.01	91.10 ± 5.30	92.02 ± 5.11
DPPC	5.50 ± 0.00	15.06 ± 0.59	25.17 ± 0.93	33.53 ± 1.28	42.88 ± 3.10	54.99 ± 3.80	67.24 ± 3.81	78.16 ± 2.59	91.96 ± 3.02	92.00 ± 4.16	92.07 ± 4.58	92.24 ± 5.32
CA	10.334 ± 1.12	24.24 ± 1.55	54.88 ± 2.85	71.98 ± 3.23	92.53 ± 6.34	92.88 ± 5.12	92.90 ± 5.12	93.00 ± 5.01	93.13 ± 5.22	93.25 ± 5.32	93.27 ± 5.31	93.30 ± 5.33

**Table 6:** Kinetic parameters of caffeic acid release (range 0.5–5 h).

Formulas	Model	Parameter	Value	R^2^
CNA	Higuchi	kH (%.h^−1/2^)	30.1	0.987
DPPC	Higuchi	kH (%.h^−1/2^)	30.63	0.985
CNA	Korsmeyer–Peppas	N	1.07	0.975
DPPC	Korsmeyer–Peppas	N	0.94	0.979
CNA	First-order	k_1_ (h^−1^)	0.145	0.987
DPPC	First-order	k_1_ (h^−1^)	0.154	0.985
CNA	Zero-order	k_0_ (%/h)	10.29	0.992
DPPC	Zero-order	k_0_ (%/h)	10.92	0.995

Legend: R^2^ represents the correlation coefficient obtained from linear regression analysis.

## Data Availability

The original contributions presented in this study are included in the article. Further inquiries can be directed to the corresponding author.

## Supplementary Materials

The following supporting information can be downloaded at: https://www.mdpi.com/article/10.3390/antiox15040424/s1, Figure S1. Profile images on selected areas (left side) and roughness histogram (right side) of the CAN-50 sample on day 1, 2, 15 and 30 (from top to bottom); Figure S2. Profile images on selected areas (left side) and roughness histogram (right side) of the eCAN sample on day 1, 2, 15 and 30 (from top to bottom); Figure S3. Profile images on selected areas (left side) and roughness histogram (right side) of the DPPC-50 sample on day 1, 2, 15 and 30 (from top to bottom); Figure S4. Profile images on selected areas (left side) and roughness histogram (right side) of the eDPPC sample on day 1, 2, 15 and 30 (from top to bottom).
