# Supplementary material for: Pharmacological Properties and the Impact of Caffeic Acid-Entrapped Liposomes on Triple-Negative Breast Cancer Cell Lines Exposed to Doxorubicin
Source: Antioxidants (Basel). 2026 Mar 27;15(4):424. doi: 10.3390/antiox15040424 (PMC13113547; doi:10.3390/antiox15040424)
Supplement: Supplementary file 1 [file antioxidants-15-00424-s001.zip › antioxidants-4167275-supplementary.pdf]

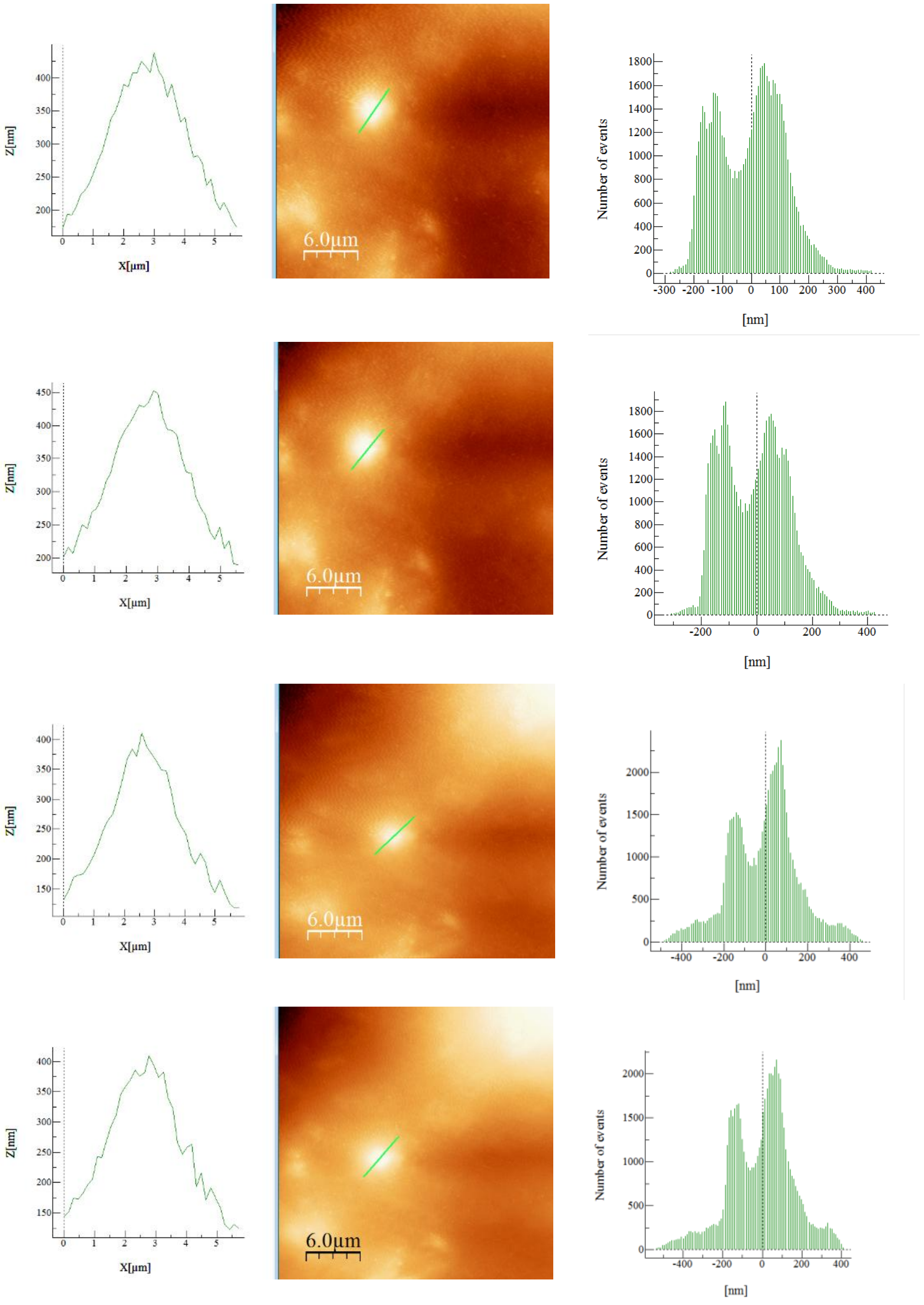

Figure S1. Profile images on selected areas (left side) and roughness histogram (right side) of the CAN-50 sample on day 1, 2, 15 and 30 (from top to bottom)

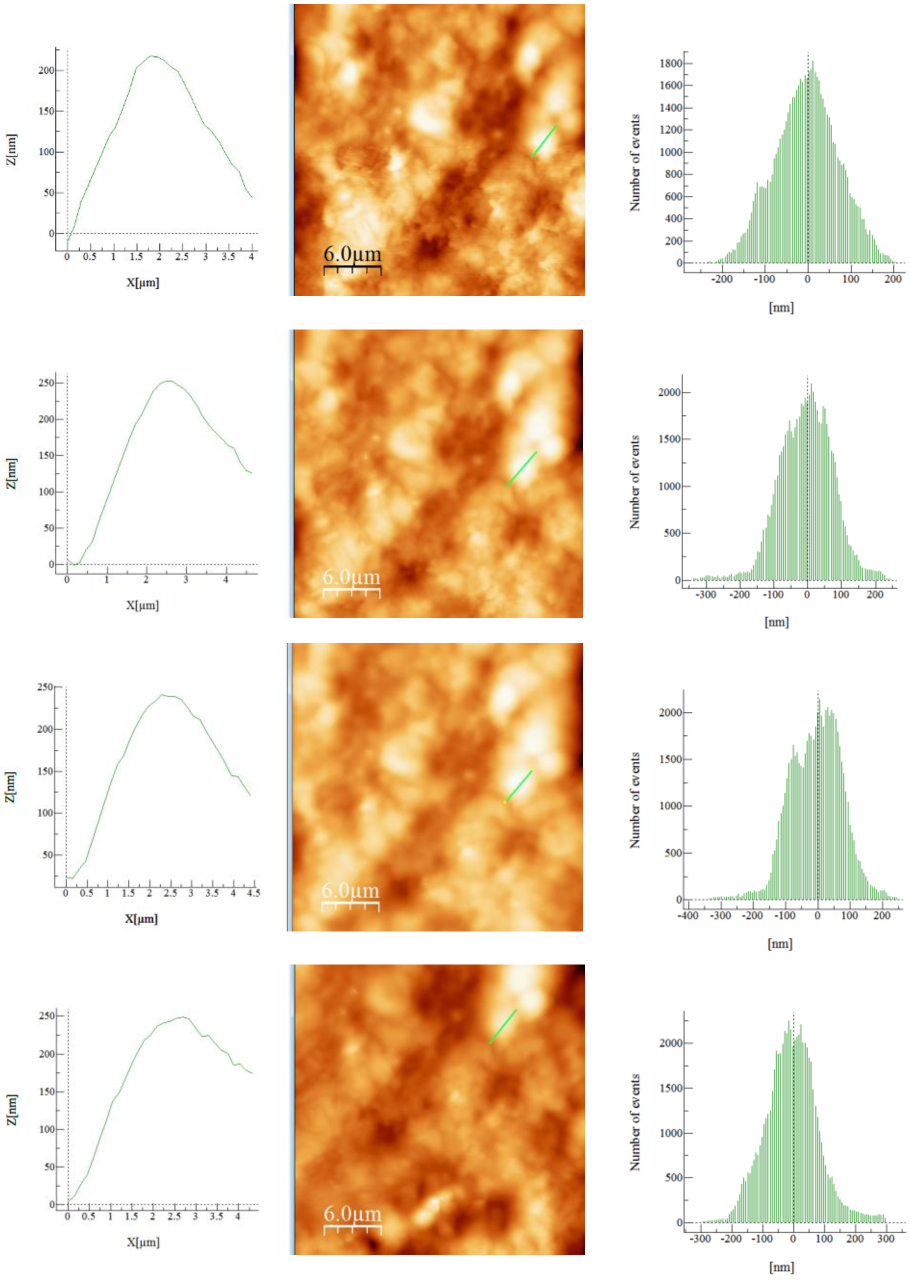

Figure S2. Profile images on selected areas (left side) and roughness histogram (right side) of the eCAN sample on day 1, 2, 15 and 30 (from top to bottom)

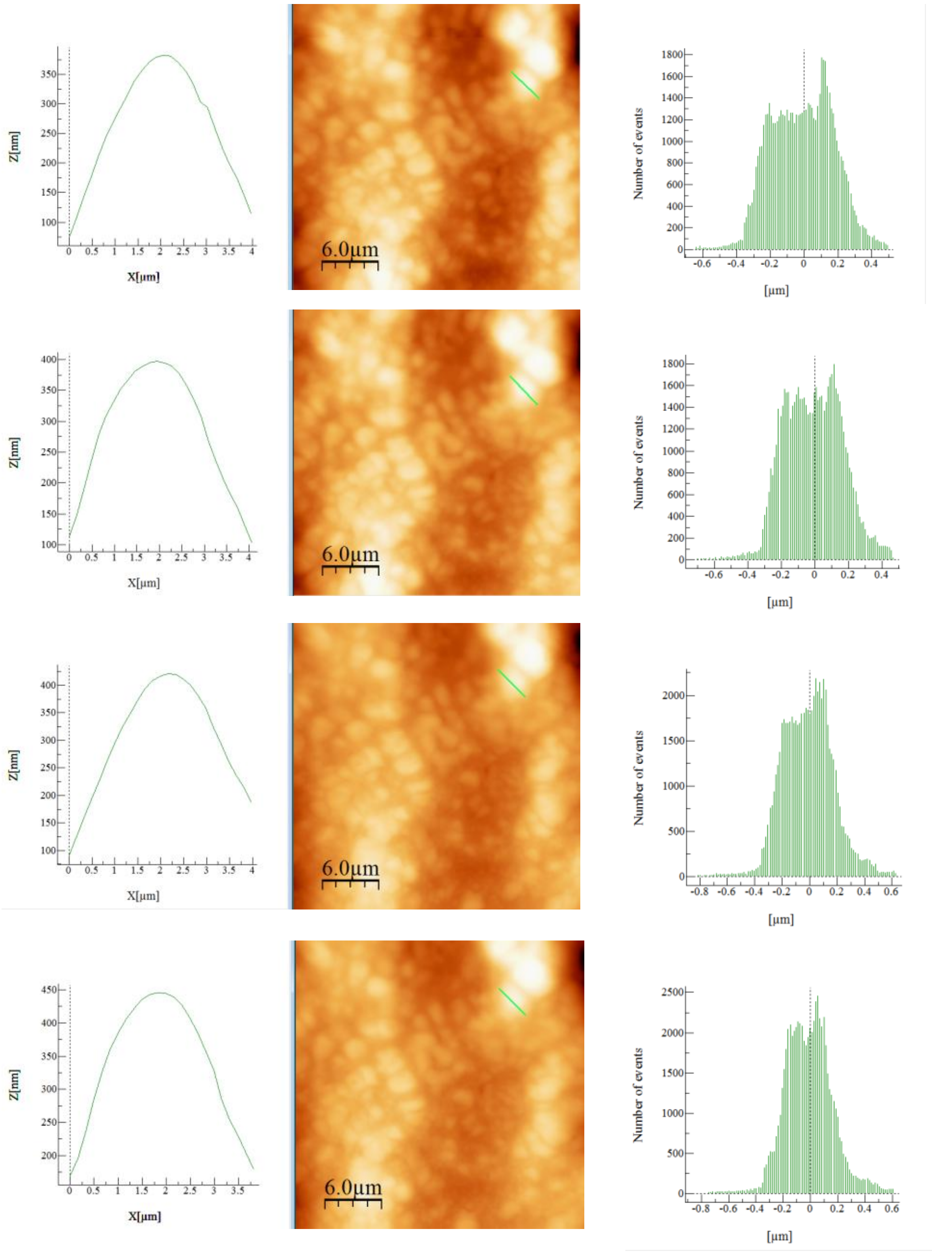

Figure S3. Profile images on selected areas (left side) and roughness histogram (right side) of the DPPC-50 sample on day 1, 2, 15 and 30 (from top to bottom)

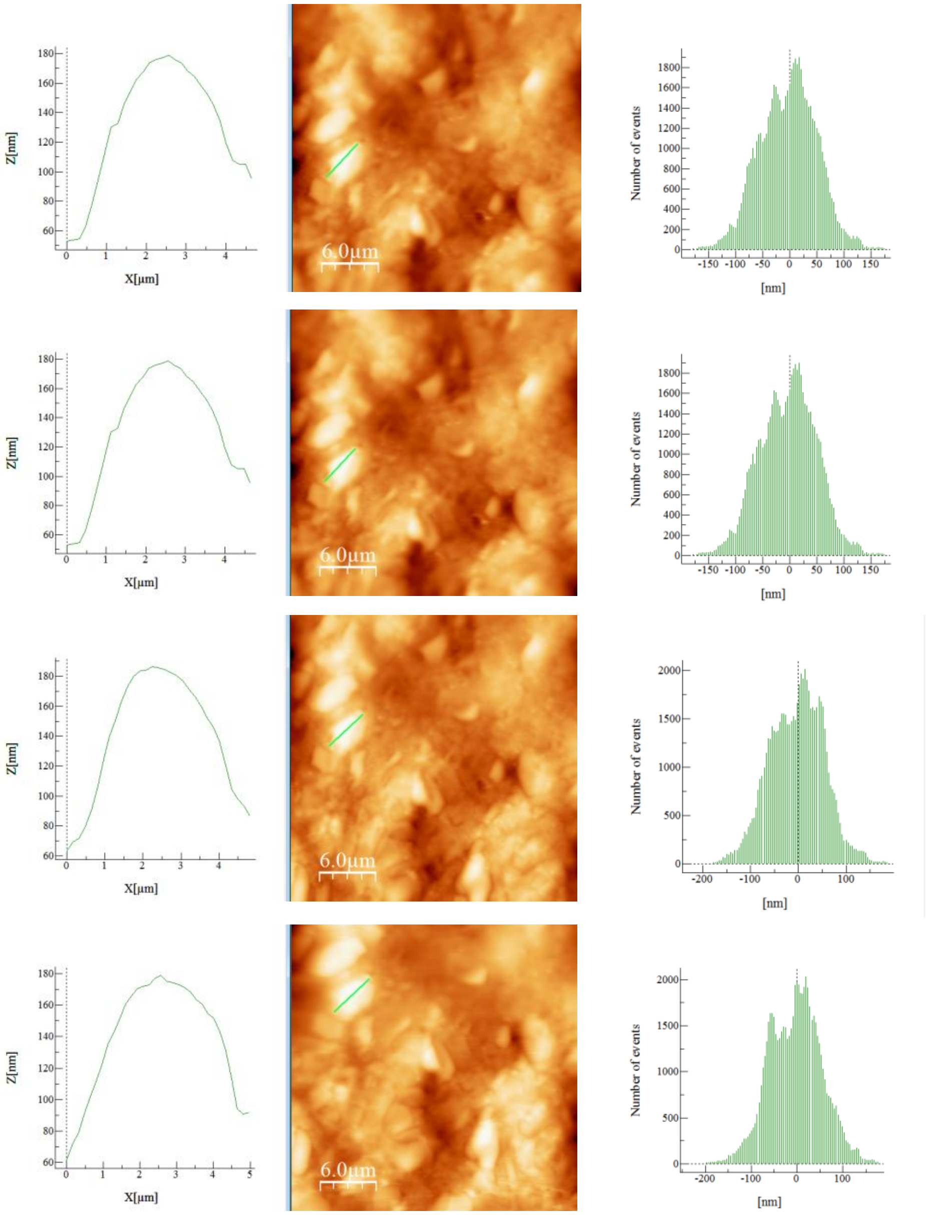

Figure S4. Profile images on selected areas (left side) and roughness histogram (right side) of the eDPPC sample on day 1, 2, 15 and 30 (from top to bottom)
